# Chronic corticosterone disrupts the circadian rhythm of *CRH* expression and m^6^A RNA methylation in the chicken hypothalamus

**DOI:** 10.1186/s40104-022-00677-4

**Published:** 2022-03-08

**Authors:** Yang Yang, Wanwan Han, Aijia Zhang, Mindie Zhao, Wei Cong, Yimin Jia, Deyun Wang, Ruqian Zhao

**Affiliations:** 1 MOE Joint International Research Laboratory of Animal Health & Food Safety, Institute of Immunology, Nanjing Agricultural University, Nanjing, 210095 People’s Republic of China; 2 Key Laboratory of Animal Physiology & Biochemistry, College of Veterinary Medicine, Nanjing Agricultural University, Nanjing, 210095 People’s Republic of China; 3 Institute of Traditional Chinese Veterinary Medicine, College of Veterinary Medicine, Nanjing Agricultural University, Nanjing, 210095 People’s Republic of China

**Keywords:** Chronic corticosterone exposure, Circadian rhythms, CRH, Hypothalamus, m^6^A

## Abstract

**Background:**

Corticotropin-releasing hormone (CRH), the major secretagogue of the hypothalamic-pituitary-adrenal (HPA) axis, is intricately intertwined with the clock genes to regulate the circadian rhythm of various body functions. N6-methyladenosine (m^6^A) RNA methylation is involved in the regulation of circadian rhythm, yet it remains unknown whether CRH expression and m^6^A modification oscillate with the clock genes in chicken hypothalamus and how the circadian rhythms change under chronic stress.

**Results:**

Chronic exposure to corticosterone (CORT) eliminated the diurnal patterns of plasma CORT and melatonin levels in the chicken. The circadian rhythms of clock genes in hippocampus, hypothalamus and pituitary are all disturbed to different extent in CORT-treated chickens. The most striking changes occur in hypothalamus in which the diurnal fluctuation of *CRH* mRNA is flattened, together with mRNA of other feeding-related neuropeptides. Interestingly, hypothalamic m^6^A level oscillates in an opposite pattern to *CRH mRNA*, with lowest m^6^A level after midnight (ZT18) corresponding to the peak of *CRH* mRNA before dawn (ZT22). CORT diminished the circadian rhythm of m^6^A methylation with significantly increased level at night. Further site-specific m^6^A analysis on 3’UTR of *CRH* mRNA indicates that higher m^6^A on 3’UTR of *CRH* mRNA coincides with lower *CRH* mRNA at night (ZT18 and ZT22).

**Conclusions:**

Our results indicate that chronic stress disrupts the circadian rhythms of CRH expression in hypothalamus, leading to dysfunction of HPA axis in the chicken. RNA m^6^A modification is involved in the regulation of circadian rhythms in chicken hypothalamus under both basal and chronic stress conditions.

**Graphical Abstract:**

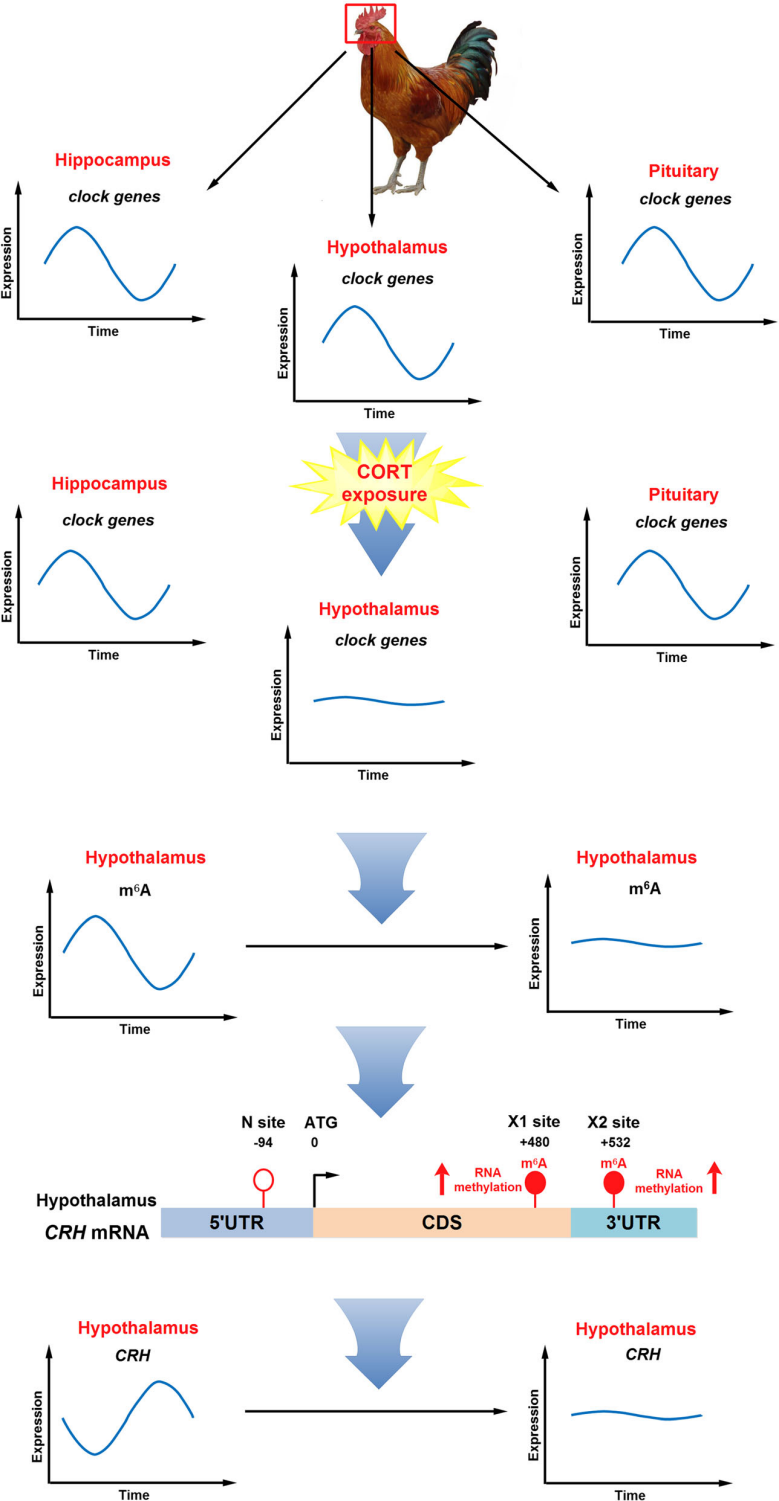

**Supplementary Information:**

The online version contains supplementary material available at 10.1186/s40104-022-00677-4.

## Introduction

The hypothalamus plays an important role in the regulation of hypothalamic-pituitary-adrenal (HPA) axis [1], feeding behavior [2], and circadian rhythm [3]. Corticotropin-releasing hormone (CRH) released from hypothalamus stimulates pituitary ACTH secretion to modulate the activity of HPA axis [4]. Moreover, CRH is involved in the regulation of food intake [5] via interacting with appetite inhibiting proopiomelanocortin (POMC)/cocaine amphetamine-regulated transcript (CART) neurons and the appetite-inducing neuropeptide Y (NPY) and agouti-related protein (AgRP) neurons [6]. Both the HPA axis activity and the feeding behavior exhibit diurnal patterns, which indicates complex interactive networks with the master clock located in the suprachiasmatic nucleus (SCN) of the hypothalamus [7]. SCN can projects to the pineal gland that secretes the hormone melatonin [8]. The core molecular clock consists of a transcriptional-translational autoregulatory “loop” with a positive arm and a negative arm [9]. The clock and bmal1 genes and their protein products comprise the positive arm, while the period (*PER1*, *PER2*, and *PER3*) and cryptochrome (*CRY1*, *CRY2*) genes and their protein products comprise the negative arm. An early research reported that the HPA system in the chicken displays a circadian rhythm [10]. Studies in mice indicate that CRH is intricately intertwined with the clock genes to regulate the circadian rhythm of various body functions [11]. However, the circadian rhythm of CRH expression in chicken hypothalamus has not been characterized.

CRH binds to CRH receptors type 1 (CRHR1) and type 2 (CRHR2) in the pituitary, causing the production and secretion of adrenocorticotropic hormone (ACTH) [12] to regulate the stress response of the body through corticosterone (CORT) synthesis and secretion from adrenal cortex [13, 14]. CORT exerts a negative feedback regulation on CRH synthesis and secretion through its receptor, glucocorticoid receptor (GR), at different levels including hippocampus and hypothalamus [15]. Chronically elevated circulating CORT has detrimental physiological and cognitive effects [16], including HPA axis dysfunction and neuroinflammation [17], as well as depressive and anxiety-like behaviors in SD rats [18]. In addition, chronic stress causes irregular expression of circadian regulatory clock genes in mouse hippocampus [19], hypothalamus SCN [20] and pituitary [21]. However, it remains unknown how chronic CORT exposure affects the circadian rhythms of clock-related genes in the chicken brain, and how it is related to the circadian rhythm of CRH in hypothalamus.

N^6^-methyladenosine (m^6^A) is the most prevalent modification in RNAs, which plays an important role in RNA splicing, degradation, and translation [22, 23]. M^6^A level is finely balanced through interplay among m^6^A methyltransferases (“writers”, such as METTL3, METTL14 and WTAP), demethylases (“erasers”, such as fat mass and obesity-associated gene *FTO* and *ALKBH5*), and binding proteins (“readers”, such as YTHDF1, YTHDF2 and YTHDF3) [24]. Chronic stress is reported to modulate m^6^A modification in the brain [25]. For instance, heat exposure for 6 h increases m^6^A RNA methylation levels in the hypothalamus of 3-day-old chickens [26]. Yet, chronic CORT treatment reduces the m^6^A methylation in chicken liver [27]. Moreover, m^6^A methylation has been reported to have circadian rhythm [28]. Clock gene *CRY1/2* knockout mice show significantly lower m^6^A level and lost the circadian rhythm of m^6^A level in RNA [28]. However, studies in the chicken are scarce. Questions remain regarding whether m^6^A modification in chicken hypothalamus show a circadian rhythm, whether the m^6^A rhythmicity, if any, is interrupted by chronic CORT exposure, and whether m^6^A is involved in the regulation of CRH expression in chicken hypothalamus.

Therefore, the objectives of the present study were, firstly, to elaborate the effects of chronic CORT exposure on circadian rhythms of clock-related genes in different brain areas including hippocampus, hypothalamus and pituitary; secondly, to delineate the circadian rhythms of *CRH* mRNA expression and m^6^A methylation in chicken hypothalamus, and to reveal their responses to chronic CORT exposure; and thirdly, to investigate the possible link between m^6^A modification and *CRH* mRNA expression in chicken hypothalamus.

## Materials and methods

### Ethics statement

The experimental protocol was approved by the Animal Ethics Committee of Nanjing Agricultural University. The project number is 31972638. The sampling procedures complied with the “Guidelines on Ethical Treatment of Experimental Animals” (2006) No.398 set by the Ministry of Science and Technology, China.

### Animals and experimental design

Seventy 45-day-old male bantam chickens were purchased from Changzhou Lihua Livestock and Poultry Co., Ltd. After a three-day adaption, chickens were randomly divided into vehicle (CON) and corticosterone (CORT) group. Light regime was 12 light: 12 dark, with light on at 07:00 as zeitgeber time 0 (ZT0) and off at 19:00 as ZT12. Food and water were provided ad libitum. CORT (Sigma-Aldrich, St Louis, USA) was sonicated in saline with 0.1% Tween 80 and 0.2% DMSO until dissolved and protected from light. Chickens were injected (twice per day, 9:00–10:00 and 18:00–19:00) intraperitoneally with vehicle or CORT (4 mg/kg BW), according to previous publication [29], for 11 consecutive days. Daily food consumption and body weight were recorded every other day. By the end of the treatment, the chickens were sacrificed at the indicated time points (ZT2, ZT6, ZT10, ZT14, ZT18 and ZT22). Chickens were anesthetized with sodium pentobarbital and the brain was quickly separated from the skull. Hippocampus [30] and hypothalamus [31] were dissected as described in previous publications according to the chicken brain atlas [32]. Pituitary was removed as previously described [33]. Tissues collected were frozen immediately in liquid nitrogen and stored at − 80 °C until use.

### Measurement of corticosterone and melatonin

Corticosterone concentration was determined by Enzyme Immunoassay (EIA) kit (No. ADI-900-097, Enzo, Farmingdale, NY, USA) following the manufacturer’s instructions. Serum melatonin levels were measured using Chicken MT (Melatonin) ELISA Kit (MM-34278O1, ImmunoWay Biotechnology, Plano, TX, USA) following the manufacturer’s instructions.

### RNA isolation and real-time PCR

High quality total RNA was isolated from hippocampus, hypothalamus and pituitary using Trizol reagents (Invitrogen, Carlsbad, CA, USA). One microgram of RNA was reverse-transcribed according to the manufacturer’s protocol (Vazyme Biotech, Nanjing, Jiangsu, China). Four microliter cDNA was diluted (1:25) and then used for real-time PCR in a QuantStudioTM 6 Flex Real-Time PCR System (Applied Biosystems, Foster City, CA, USA). Peptidylprolyl isomerase A (*PPIA*) was used as an internal control to normalize the technical variations. Data were analyzed using the method of 2^-ΔΔCT^ and presented relative to the CON group. All primers (Table 1) were synthesized by Suzhou GENEWIZ Biological Technology Co., Ltd. (Suzhou, Jiangsu, China).

**Table 1:** The primers sequences for RT-PCR and SELECT

Target genes	Primer sequences (5′ to 3′)	
*CLOCK*	F: GATCACAGGGCACCTCCAATA	R: CTAGTTCTCGCCGCCTTTCT
*B1AML1*	F: GTAGACCAGAGGGCGACAG	R: ATGAAACTGAACCAGCGACTC
*CRY1*	F: GATGTGGCTATCCTGTAGTTCCT	R: GCTGCTGGTAGATTTGTTTCAT
*CRY2*	F: GCACGGCTGGATAAACACT	R: AAATAAGCGGCAGGACAAA
*PER2*	F: ATGAAACGAGCCATCCCG	R: CAGTTGTCGTGATTTTGCCTA
*PER3*	F: CAGTGCCTTTGTTGGGTTAC	R: GATGGATTCACAAAACTGGAC
*CRH*	F: CTCCCTGGGCCTGGCTTT	R: CCTCACTTCCCGATGATT
*CRHR1*	F: CACAGCCTTCATCCTACGCA	R: CGGAGCTTGTCGGTGGAATA
*CRHR2*	F: TCTTTCCTGGGCTTTCACGG	R: ATTGAAGAACTCCGGGCAGG
*NPY*	F: ACTCGGCTCTGAGGCACT	R: GGTCTTCAAACCGGGATC
*FTO*	F: TCACCAAGGCGACCTCTACT	R: GCTGAACCGAGGTGAAAAGC
*METTL3*	F: ATCCTGGAGCTGCTCAACAC	R: AGATTCGTCCGTGTGCTTGT
*METTL14*	F: ATTCGACCAGGATGGCTGAC	R: GACTTGGGTGGTGGTGACTT
*YTHDF1*	F: AACAACCAGCTCCGACACAT	R: GATTCTGACGTTCCTTCCGC
*YTHDF2*	F: AAGGCCAAGGCAACAAAGTG	R: ATATGCATTGTTCGGCCGGG
*YTHDF3*	F: CGTAATAGGGGTGTGGGCTTC	R: CACTTCCACACCAGAAGGTGA
*PPIA*	F: TTACGGGGAGAAGTTTGCCG	R: TGGTGATCTGCTTGCTCGTC
*SELECT*		
*CRH N site*	F: tagccagtaccgtagtgcgtgGGCGCGCAGCGCGGCCGCTG R: CCCGGTGCTGAAACGCGGCCcagaggctgagtcgctgcat	
*CRH X1 site*	F: tagccagtaccgtagtgcgtgTTCCCGATGATTTCCATCAG R: TTCCTGTTGCTGTGGGCTTGcagaggctgagtcgctgcat	
*CRH X2 site*	F: tagccagtaccgtagtgcgtgCTCTGGTGCTGACCGCGGGG R: CCCTTTGGCACGGCGCGGGGcagaggctgagtcgctgcat	

### Analysis of mRNA m^6^A methylation by dot-blotting assay

Dot-blot analysis of mRNA m^6^A methylation was performed following a published procedure with minor modifications [34]. Briefly, total RNAs were isolated using the Trizol method and mRNAs were enriched by using GenElute™ mRNA Miniprep Kit (Sigma, Burlington, NJ, USA). The concentration and purity of mRNAs were measured by NanoDrop 2000. The mRNAs were denatured by heating at 95 °C for 5 min, followed by chilling on ice immediately. Next, the mRNA (100 ng) was spotted directly onto the positively charged nylon membrane (GE Healthcare, Pittsburgh, PA, USA) and air dried for 5 min. The membrane was then UV crosslinked in Ultraviolet Crosslinker, blocked with 5% of nonfat milk in TBST, and then incubated with anti-m^6^A antibody overnight at 4 °C. HRP-conjugated anti-rabbit IgG secondary antibody was added to the membrane for 2 h at room temperature with gentle shaking and then developed with enhanced chemiluminescence. Methylene blue staining was used to verify that equal amount mRNA spotted on the membrane.

### Single-base elongation and ligation-based qPCR amplification method (SELECT) assay

The SELECT assay for monitoring site-specific m^6^A levels in the 3′UTR of *CRH* mRNA was performed as described previously [35]. In brief, total RNA (2 μg) was mixed with 1 μL of 100 μmol/L dNTP (NEB, Ipswich, MA, USA), 2 μL of CutSmart buffer (NEB, Ipswich, MA, USA), and 2 μL each of 400 nmol/L up and down DNA probes (Table 1). The total volume was adjusted to 17 μL with water. The DNA probes and RNA were annealed by incubating the mixture with a temperature gradient of 90 °C for 1 min, 80 °C for 1 min, 70 °C for 1 min, 60 °C for 1 min, 50 °C for 1 min, and 40 °C for 6 min. To the mixture was then added a 3 μL solution containing 0.01 U *Bst* 2.0 DNA polymerase, 0.5 U SplintR ligase, and 10 nmol ATP. After incubating at 40 °C for 20 min and then at 80 °C for 20 min, an aliquot (2 μL) of the reaction mixture was taken out for real-time qPCR analysis to quantify template abundance.

### Statistical analysis

Data are presented as the mean ± standard error of the mean (SEM). The mRNA levels of clock-related genes and melatonin contents were analyzed using one-way analysis of variance (one-way ANOVA) with IBM SPSS Statistics 20 software (United States) to test the statistical significance of the differences among the six daily time points and confirm the daily variation (*P* ≤ 0.05), as the premise of cosinor analysis. To determine the circadian rhythmicity of each clock-related gene profile, the mRNA levels of clock-related genes, as well as CORT and melatonin levels were analyzed separately using MATLAB 7.0 (MathWorks Inc., USA) based on unimodal cosinor regression [*y* = A + (B × cos (2π(*x* − C)/24))]. A, B and C represent the mesor, amplitude and acrophase, respectively. The results of regression analysis were considered significant at *P* ≤ 0.05, which was calculated using the number of samples, R^2^ values and the number of predictors (mesor, amplitude and acrophase) from http://www.danielsoper.com/statcalc3/calc.aspx?i1/415 [36]. Differences of the mesor, amplitude and acrophase between CON and CORT group were tested by one-way ANOVA followed by Fisher’s least significant difference (LSD) post hoc test, considering *P* ≤ 0.05 to be significant.

## Results

### Effect of chronic CORT exposure on body weight, food intake, plasma CORT and melatonin concentration

**Fig. 1:**
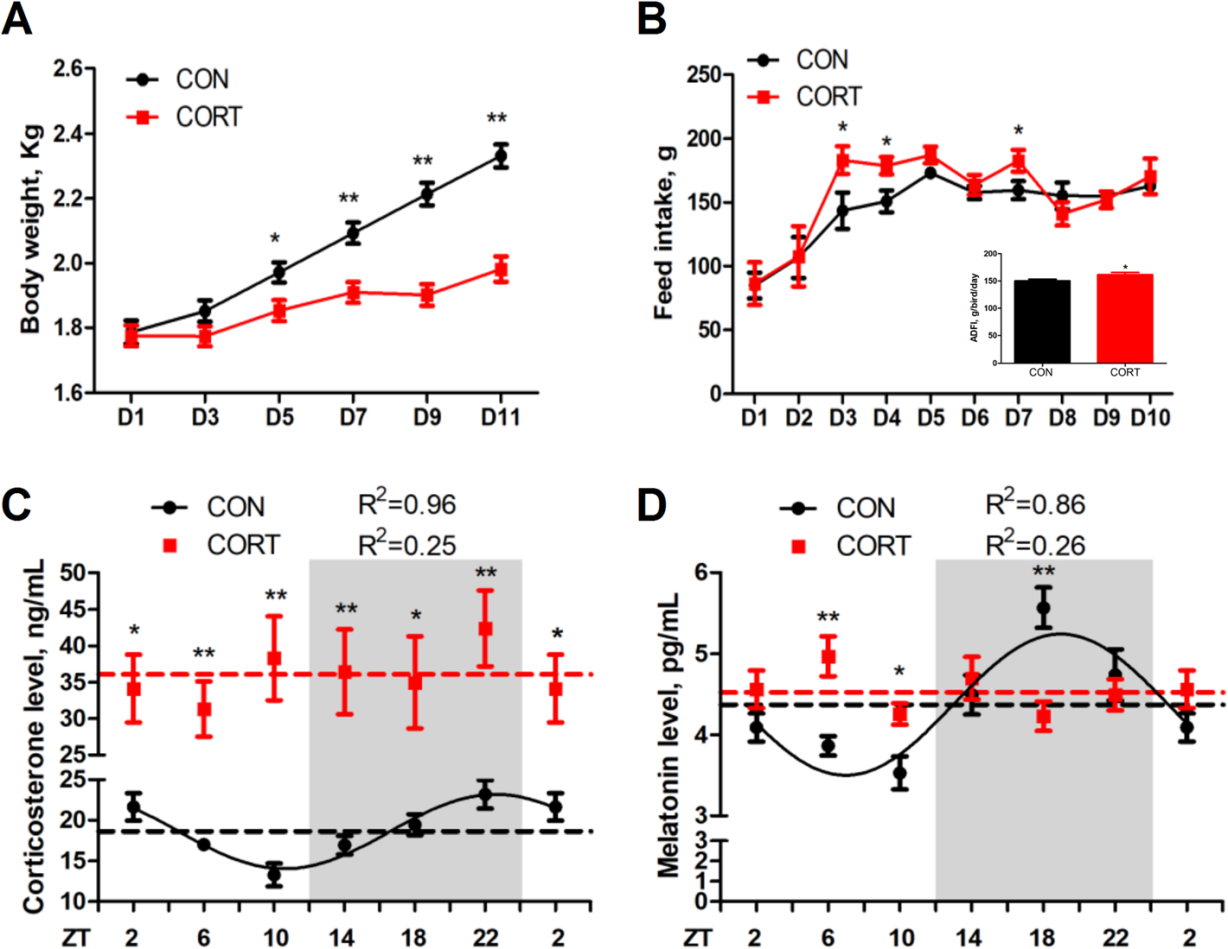
Effect of chronic CORT exposure on body weight, food intake, plasma CORT and melatonin concentration. (**A**) Body weight (*n* = 6); (**B**) Feed intake (*n* = 6) and average daily feed intake (*n* = 10); (**C**) Plasma corticosterone content; (**D**) Plasma melatonin content. The curves represent the 24-hour period determined by cosinor analysis. *n* = 6 chickens per time point. Data from CT2 are double-plotted. *R*^2^ values represent the degree of fitting. Values are mean ± SEM, *P < 0.05, ** *P* < 0.01, compared with control

Chronic CORT exposure leads to growth retardation, with significantly lower body weight, as compared with their control counterparts, from the 5th day of CORT injection (D5) to D11 (Fig.1A). Interestingly, the feed intake was significantly increased on D3, D4 and D7, leading to significantly increased average daily feed intake (Fig.1B). Both CORT (Fig. 1C) and melatonin (Fig. 1D) levels in plasma exhibited diurnal pattern in CON group (*P* < 0.05, one-way ANOVA), which was eliminated in CORT group. The mesors of CORT level were significantly elevated (*P* < 0.01) by CORT injection, while the mesors of melatonin level did not change (Table 2).

**Table 2:** Circadian rhythm parameters of CORT and melatonin levels in plasma, as determined by cosinor analyses

Index	Group	CORT	Melatonin
Mesor	CON	18.62 ± 0.26	4.37 ± 0.12
	CORT	36.10 ± 1.45**	4.53 ± 0.10
Amplitude	CON	3.13 ± 0.37	0.87 ± 0.17
	CORT	ND	ND
Acrophase, h	CON	23.18 ± 0.46	18.97 ± 0.68
	CORT	ND	ND

Values are means ± SEM. **P < 0.01, compared with CON group. ND represents not determined as there was no circadian rhythm

### Effect of chronic CORT exposure on the circadian rhythm of clock genes in hippocampus, hypothalamus, and pituitary

**Fig. 2:**
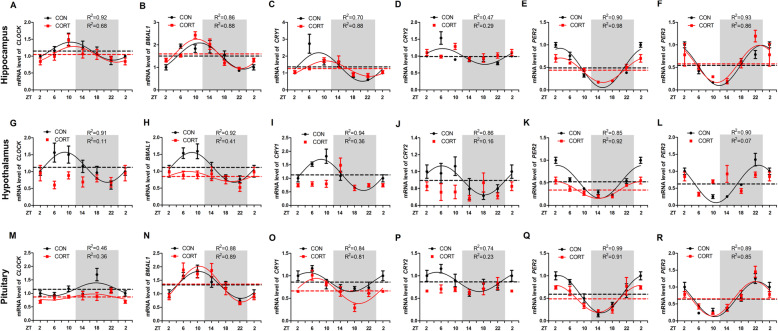
Effect of chronic CORT exposure on the circadian rhythm parameters of clock genes in chicken hippocampus, hypothalamus and pituitary. (**A-F**) The circadian rhythms of clock gene mRNA expression in chicken hippocampus. (**A**) CLOCK gene; (**B**) *BMAL1* gene; (**C**) *CRY1* gene; (**D**) *CRY2* gene; (**E**) *PER2* gene; (**F**) *PER3* gene. (**G-L**) The circadian rhythms of clock gene mRNA expression in chicken hypothalamus. (**G**) CLOCK gene; (**H**) *BMAL1* gene; (**I**) *CRY1* gene; (**J**) *CRY2* gene; (**K**) *PER2* gene; (**L**) *PER3* gene. (**M-R**) The circadian rhythms of clock gene mRNA expression in chicken pituitary. (**M**) CLOCK gene; (**N**) *BMAL1* gene; (**O**) *CRY1* gene; (**P**) *CRY2* gene; (**Q**) *PER2* gene; (**R**) *PER3* gene. The relative mRNA levels of clock gene are normalized to PPIA. The data markers in the graphs indicate the clock gene mRNA expression levels, and the results are expressed as the mean ± SEM. The curves represent the 24-h period determined by cosinor analysis. *n* = 6 chickens per time point. Data from CT2 are double-plotted. *R*^2^ values represent the degree of fitting

All the 6 clock genes were expressed in hippocampus (Fig. 2A-F), hypothalamus (Fig. 2G-L), and pituitary (Fig. 2M-R), in gene- and region-specific rhythmic patterns. In CON group, *BMAL1*, *PER2* and *PER3* showed more pronounced circadian pattern among 6 clock genes (*P* < 0.05, one-way ANOVA), regardless of the region. Among 3 brain regions, hypothalamus displayed more clearly circadian patterns for all the 6 clock genes (*P* < 0.05, one-way ANOVA) as shown in cosinor analysis. Chronic CORT exposure abolished or blunted the circadian rhythms of all the major clock genes in hypothalamus, while hippocampus and pituitary were less affected.

Specifically, chronic CORT exposure significantly delayed (*P* < 0.05) the acrophase of *CRY1* mRNA for 2 h (Fig. 2C and Table 3), and significantly decreased (*P* < 0.05) the amplitude of *PER2* mRNA in hippocampus (Fig. 2E and Table 3). However, chronic CORT exposure had no impact on the rhythmicity of *CLOCK* (Fig. 2A), *BMAL1* (Fig. 2B), *CRY2* (Fig. 2D) or *PER3* (Fig. 2F) mRNA expression in hippocampus (Table 3). By contrast, the circadian rhythms of *CLOCK* (Fig. 2G), *CRY1* (Fig. 2I), *CRY2* (Fig. 2J) and *PER3* (Fig. 2L) mRNA in hypothalamus were lost in CORT group (Table 4). Meanwhile, the mesor and amplitude of *BMAL1* (Fig. 2H) and *PER2* (Fig. 2K) mRNA were significantly decreased (*P* < 0.05) in CORT group (Table 4). In pituitary, chronic CORT exposure significantly decreased (*P* < 0.05) the mesor of *CLOCK* (Fig. 2M) and *CRY1* (Fig.2O) mRNA (Table 5). However, chronic CORT exposure had no impact on the rhythmicity of all the clock genes except *CRY2* (Fig. 2P, Table 5).

**Table 3:** Circadian rhythm parameters of all clock genes in hippocampus, as determined by cosinor analyses

Index	Group	*CLOCK*	*BMAL1*	*CRY1*	*CRY2*	*PER2*	*PER3*
Mesor	CON	1.15 ± 0.03	1.50 ± 0.09	1.36 ± 0.18	0.99 ± 0.08	0.49 ± 0.05	0.54 ± 0.04
	CORT	1.05 ± 0.06	1.60 ± 0.08	1.26 ± 0.06	1.05 ± 0.05	0.44 ± 0.05	0.58 ± 0.06
Amplitude	CON	0.27 ± 0.04	0.58 ± 0.12	0.86 ± 0.27	ND	0.44 ± 0.07	0.45 ± 0.06
	CORT	0.23 ± 0.08	0.65 ± 0.12	0.45 ± 0.27	0.09 ± 0.12	0.27 ± 0.02*	0.41 ± 0.08
Acrophase, h	CON	10.64 ± 0.55	11.14 ± 0.79	8.53 ± 0.28	5.89 ± 1.87	3.27 ± 0.69	23.80 ± 0.56
	CORT	11.51 ± 1.35	11.43 ± 0.69	10.51 ± 0.70*	ND	3.37 ± 0.32	23.13 ± 0.78

Values are means ± SEM. *P < 0.05, **P < 0.01, compared with CON group. ND represents not determined as there was no circadian rhythm

**Table 4:** Circadian rhythm parameters of all clock genes in hypothalamus, as determined by cosinor analyses

Index	Group	*CLOCK*	*BMAL1*	*CRY1*	*CRY2*	*PER2*	*PER3*
Mesor	CON	1.13 ± 0.05	1.11 ± 0.04	1.13 ± 0.05	0.90 ± 0.02	0.52 ± 0.06	0.63 ± 0.07
	CORT	ND	0.84 ± 0.06*	ND	ND	0.34 ± 0.02*	ND
Amplitude	CON	0.45 ± 0.07	0.44 ± 0.06	0.58 ± 0.07	0.17 ± 0.03	0.36 ± 0.08	0.55 ± 0.09
	CORT	ND	0.15 ± 0.09*	ND	ND	0.19 ± 0.03*	ND
Acrophase, h	CON	8.94 ± 0.53	8.43 ± 0.48	8.53 ± 0.43	6.21 ± 0.72	2.53 ± 0.92	22.88 ± 0.64
	CORT	ND	7.51 ± 2.01	ND	ND	2.03 ± 0.63	ND

Values are means ± SEM. *P < 0.05, **P < 0.01, compared with CON group. ND represents not determined as there was no circadian rhythm

**Table 5:** Circadian rhythm parameters of all clock genes in pituitary, as determined by cosinor analyses

Index	Group	*CLOCK*	*BMAL1*	*CRY1*	*CRY2*	*PER2*	*PER3*
Mesor	CON	1.15 ± 0.09	1.33 ± 0.06	0.86 ± 0.03	0.86 ± 0.05	0.59 ± 0.02	0.64 ± 0.06
	CORT	0.88 ± 0.05*	1.36 ± 0.08	0.66 ± 0.05*	ND	0.49 ± 0.04	0.65 ± 0.07
Amplitude	CON	0.24 ± 0.13	0.50 ± 0.09	0.21 ± 0.05	0.21 ± 0.06	0.41 ± 0.02	0.52 ± 0.09
	CORT	0.11 ± 0.08	0.66 ± 0.12	0.28 ± 0.07	ND	0.30 ± 0.05	0.51 ± 0.11
Acrophase, h	CON	18.25 ± 2.00	10.34 ± 0.69	5.04 ± 0.84	4.81 ± 1.15	2.87 ± 0.25	22.53 ± 0.66
	CORT	19.10 ± 2.60	10.57 ± 0.66	7.15 ± 0.84	ND	1.97 ± 0.68	21.92 ± 0.76

Values are means ± SEM. *P < 0.05, compared with CON group. ND represents not determined as there was no circadian rhythm

### Effect of chronic CORT exposure on the circadian rhythm parameters of CRH in hypothalamus and CRH receptor genes in pituitary

**Fig. 3:**
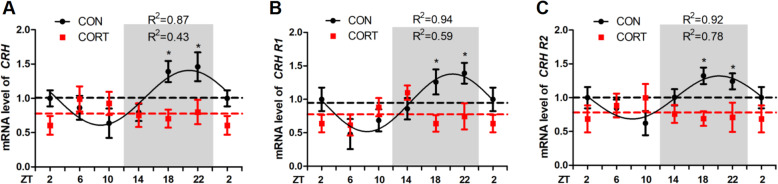
Effect of chronic CORT exposure on the circadian rhythm parameters of CRH in chicken hypothalamus and CRH receptors gene in chicken pituitary. (**A**) *CRH* gene; (**B**) *CRHR1* gene; (**C**) *CRHR2* gene. The relative mRNA levels of *CRH* and *CRH* receptors gene are normalized to PPIA. The data markers in the graphs indicate the CRH and CRH receptors gene mRNA expression levels, and the results are expressed as the mean ± SEM. The curves represent the 24-h period determined by cosinor analysis. *n* = 6 chickens per time point. Data from CT2 are double-plotted. *R*^2^ values represent the degree of fitting. *P < 0.05, compared with control

In line with the abolished rhythmicity of clock genes in hypothalamus, the circadian pattern of *CRH* mRNA (Fig. 3A) in hypothalamus was significantly diminished in CORT group, so was the rhythmic expression of *CRHR1* (Fig. 3B) and *CRHR2* (Fig. 3C) mRNA in pituitary (*P* < 0.05, one-way ANOVA). Chronic CORT exposure significantly decreased the mesor (*P* < 0.05) and amplitude (*P* < 0.01) of *CRH* mRNA in hypothalamus, as well as *CRHR1* and *CRHR2* mRNA in pituitary (Table 6). In general, chronic CORT exposure significantly abolished (*P* < 0.05) the rise of *CRH* (Fig. 3A), *CRHR1* (Fig. 3B) and *CRHR2* (Fig. 3C) mRNA expression in the dark phase after midnight at ZT18 and ZT22.

**Table 6:** Circadian rhythm parameters of *CRH* in hypothalamus, and *CRHR1*, *CRHR2* in pituitary, as determined by cosinor analyses

Index	Group	*CRH*	*CRH R1*	*CRH R2*
Mesor	CON	1.01 ± 0.05	0.95 ± 0.04	1.01 ± 0.03
	CORT	0.78 ± 0.05*	0.78 ± 0.05*	0.78 ± 0.03*
Amplitude	CON	0.40 ± 0.08	0.43 ± 0.06	0.32 ± 0.05
	CORT	0.13 ± 0.07**	0.17 ± 0.07**	0.15 ± 0.04**
Acrophase, h	CON	20.81 ± 0.65	20.35 ± 0.44	20.18 ± 0.50
	CORT	ND	ND	ND

Values are means ± SEM. *P < 0.05, **P < 0.01, compared with CON group. ND represents not determined as there was no circadian rhythm

### Effect of chronic CORT exposure on the circadian rhythm parameters of feeding and inflammation-related genes in hypothalamus

**Fig. 4:**
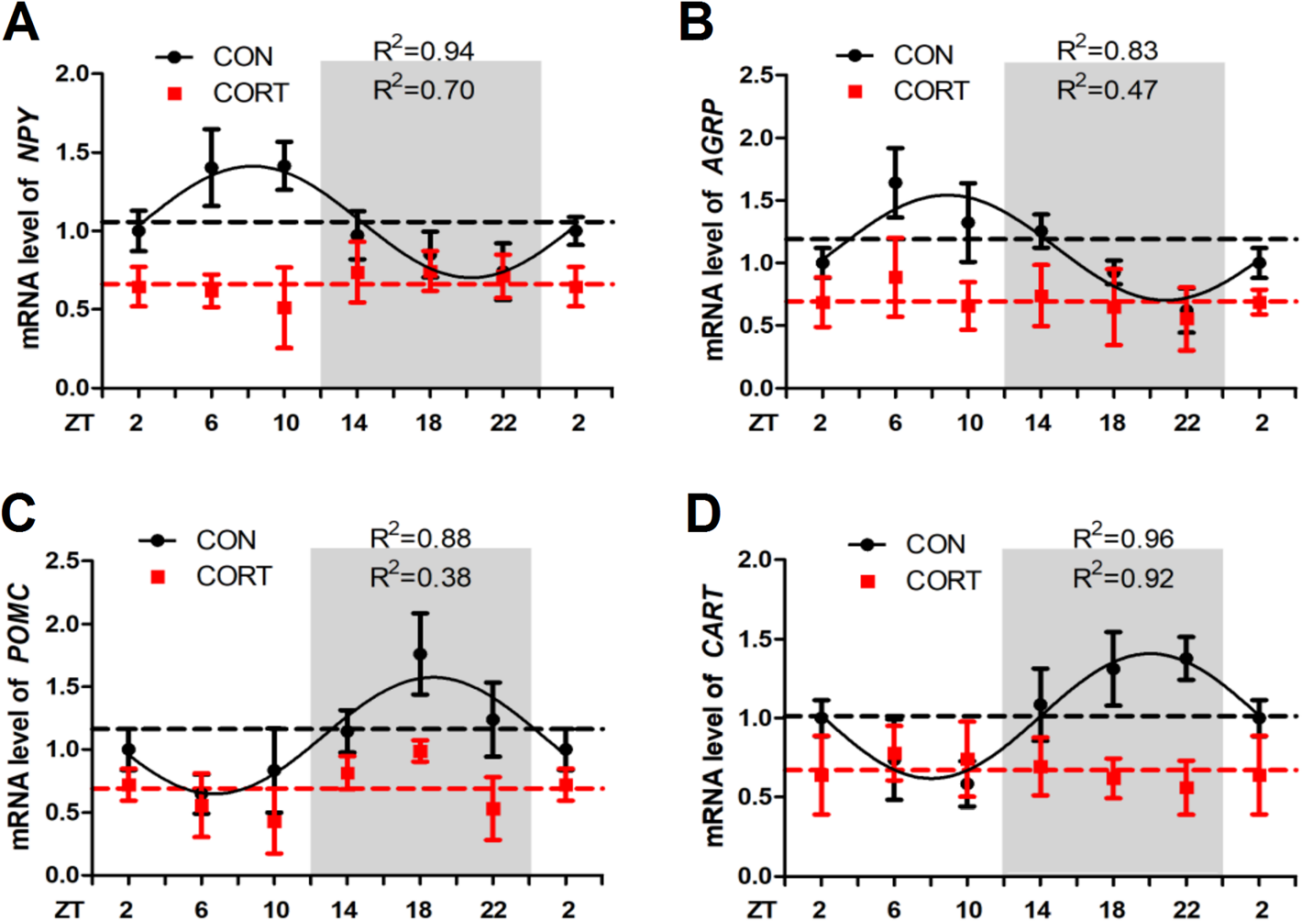
Effect of chronic CORT exposure on the circadian rhythm parameters of feeding related genes in chicken hypothalamus. The circadian rhythms of feeding related gene mRNA expression in chicken pituitary. (**A**) *NPY* gene; (**B**) *AGRP* gene; (**C**) *POMC* gene; (**D**) *CART* gene. The relative mRNA levels of feeding related gene are normalized to PPIA. The data markers in the graphs indicate the feeding related gene mRNA expression levels, and the results are expressed as the mean ± SEM. The curves represent the 24-h period determined by cosinor analysis. *n* = 6 chickens per time point. Data from CT2 are double-plotted. *R*^2^ values represent the degree of fitting

In accordance with the alterations of *CRH* mRNA, the diurnal patterns of hypothalamic *NPY* (Fig. 4A), *AGRP* (Fig. 4B), *POMC* (Fig. 4C) and *CART* (Fig. 4D) RNA expression were also eliminated in CORT group (*P* < 0.05, one-way ANOVA). The expression pattern of “the hunger genes” *NPY* and *AGRP* were opposite to that of the “the satiety genes” *POMC* and *CART*, matching the diurnal pattern of feeding behavior in the chicken. Chronic CORT exposure significantly decreased (*P* < 0.01) the mesor and amplitude of all the 4 feeding regulatory genes in hypothalamus (Table 7). In addition, chronic CORT exposure significantly increased (*P* < 0.01) the hypothalamic expression of *TNF-α*, *IL-1β* and *IL-6* mRNA, among which the circadian rhythms of *TNF-α* and *IL-6* mRNA was diminished (Additional file 1: Fig. S1).

**Table 7:** Circadian rhythm parameters of *NPY*, *AGRP*, *POMC* and *CART* in hypothalamus, as determined by cosinor analyses

Index	Group	*NPY*	*AGRP*	*POMC*	*CART*
Mesor	CON	1.06 ± 0.03	1.12 ± 0.06	1.11 ± 0.06	1.01 ± 0.03
	CORT	0.66 ± 0.02**	0.69 ± 0.03**	0.69 ± 0.07**	0.67 ± 0.01**
Amplitude	CON	0.36 ± 0.05	0.42 ± 0.10	0.46 ± 0.08	0.40 ± 0.04
	CORT	0.10 ± 0.03**	0.10 ± 0.05**	0.16 ± 0.10**	0.10 ± 0.01**
Acrophase, h	CON	8.25 ± 0.44	8.86 ± 0.77	18.72 ± 0.63	20.03 ± 0.35
	CORT	ND	ND	ND	ND

Values are means ± SEM. *P < 0.05, **P < 0.01, compared with CON group. ND represents not determined as there was no circadian rhythm

### Effect of chronic CORT exposure on the circadian rhythm parameters of m^6^A level and m^6^A related genes in hypothalamus

**Fig. 5:**
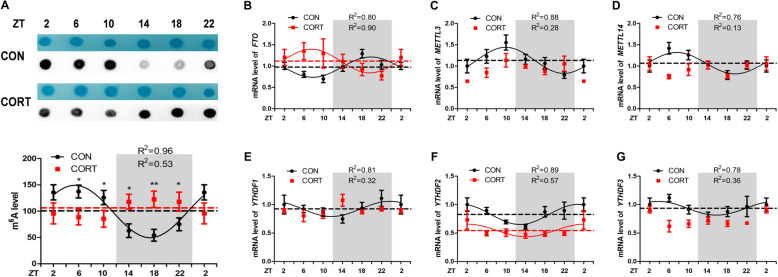
Effect of chronic CORT exposure on the circadian rhythm parameters of m^6^A level and m^6^A related genes in chicken hypothalamus. The circadian rhythms of m^6^A level and m^6^A related genes mRNA expression in chicken pituitary. (**A**) M^6^A level (*n* = 4); (**B**) *FTO* gene; (**C**) *METTL3* gene; (**D**) *METTL14* gene; (**E**) *YTHDF1* gene; (**F**) *YTHDF2* gene; (G) *YTHDF3* gene. The relative mRNA levels of m^6^A related genes are normalized to PPIA, *n* = 6 chickens per time point. The data markers in the graphs indicate the m^6^A related genes mRNA expression levels, and the results are expressed as the mean ± SEM. The curves represent the 24-h period determined by cosinor analysis. Data from CT2 are double-plotted. *R*^2^ values represent the degree of fitting. *P < 0.05, compared with control

Interestingly, the global RNA m^6^A levels (Fig. 5A) exhibited diurnal pattern in CON group (*P* < 0.05, one-way ANOVA), higher m^6^A levels were detected in light phase. Chronic CORT exposure significantly disrupted this pattern with significantly decreased (*P* < 0.05) m^6^A levels in light phase at ZT6 and ZT10, but significantly increased (*P* < 0.05) m^6^A levels in dark phase at ZT14, ZT18 and ZT22. Meanwhile, chronic CORT exposure significantly (*P* < 0.01) decreased the amplitude of m^6^A levels and delayed the acrophase of m^6^A levels for 13.48 h (Table 8). Concurrently, chronic CORT exposure significantly increased (*P* < 0.05) the mesor of *FTO* (Fig. 5B) mRNA and decreased (*P* < 0.05) the mesor of *YTHDF2* (Fig. 5F) and *YTHDF3* (Fig. 5G) mRNA in hypothalamus (Table 8).

**Table 8:** Circadian rhythm parameters of m^6^A level and m^6^A related genes in hypothalamus, as determined by cosinor analyses

Index	Group	m^6^A	*FTO*	*METTL3*	*METTL14*	*YTHDF1*	*YTHDF2*	*YTHDF3*
Mesor	CON	99.31 ± 3.52	0.97 ± 0.04	1.13 ± 0.04	1.07 ± 0.05	0.92 ± 0.02	0.83 ± 0.02	0.94 ± 0.02
	CORT	104.6 ± 1.92	1.12 ± 0.03*	0.91 ± 0.09	ND	ND	0.54 ± 0.04*	0.73 ± 0.05*
Amplitude	CON	49.76 ± 5.04	0.24 ± 0.06	0.31 ± 0.05	0.25 ± 0.07	0.13 ± 0.03	0.18 ± 0.03	0.12 ± 0.03
	CORT	20.76 ± 2.84**	0.28 ± 0.05	0.17 ± 0.07	ND	ND	0.11 ± 0.55	0.09 ± 0.06
Acrophase, h	CON	4.31 ± 0.45	19.82 ± 0.83	9.93 ± 0.67	9.58 ± 0.95	23.73 ± 0.98	0.87 ± 0.75	3.76 ± 1.10
	CORT	ND	7.56 ± 0.58**	15.10 ± 3.43*	ND	ND	2.12 ± 1.90	1.31 ± 2.87

Values are means ± SEM. *P < 0.05, **P < 0.01, compared with CON group. ND represents not determined as there was no circadian rhythm

### Effect of chronic CORT exposure on the site-specific m^6^A levels in the 3’UTR of CRH mRNA in hypothalamus

**Fig. 6:**
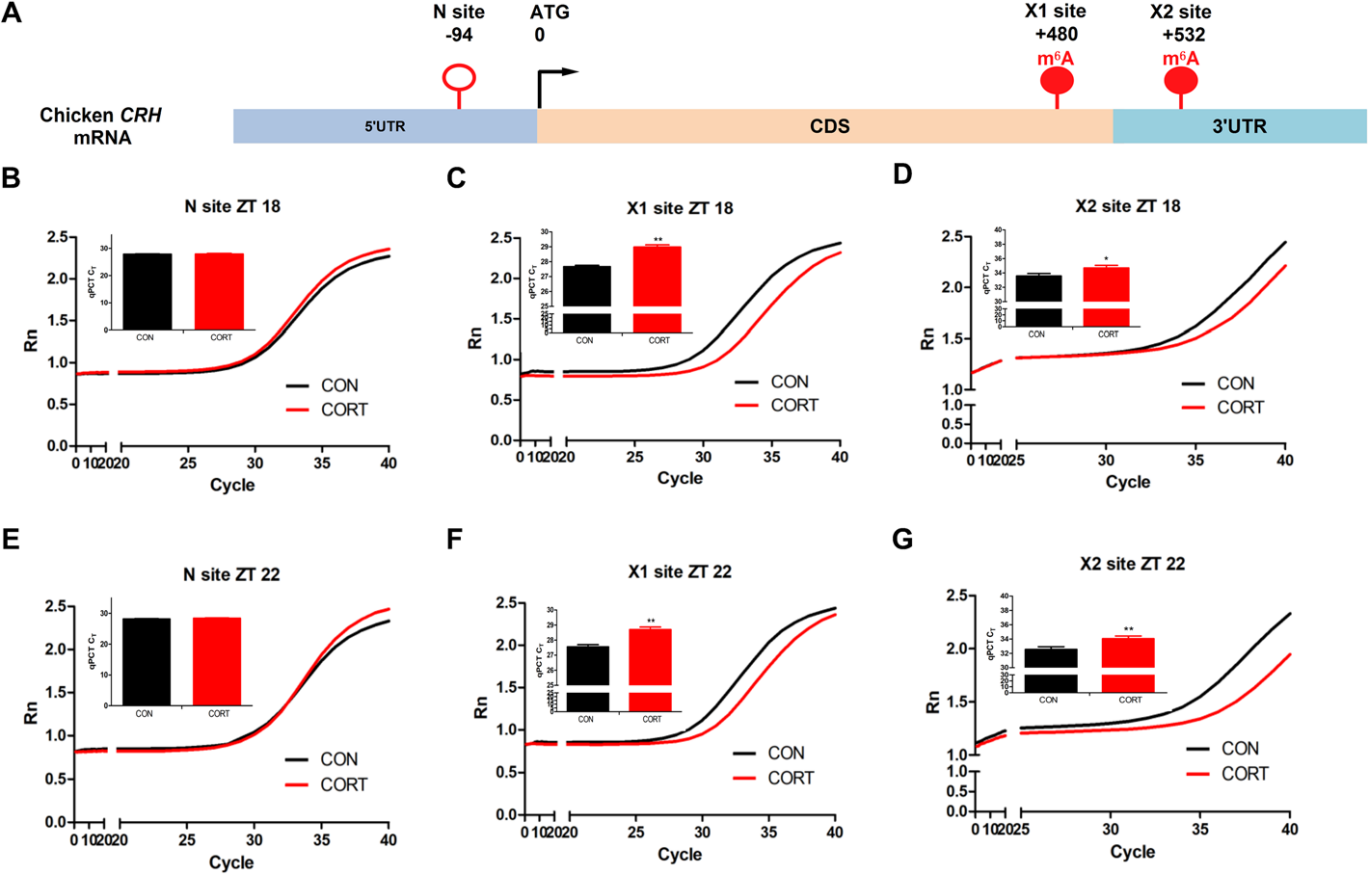
Effect of chronic CORT exposure on the site-specific m^6^A levels in the 3’UTR of *CRH* mRNA in chicken hypothalamus. Validation of m^6^A modification in CRH 3’UTR using single-base elongation and ligation-based qPCR amplification method (SELECT) when treatment with CORT in chicken hypothalamus. (**A**) Schematic graph of N, X1 and X2 site in *CRH* gene; (**B**) Amplification curve and qPCR CT value in CRH N site at ZT18; (**C**) Amplification curve and qPCR CT value in CRH X1 site at ZT18; (**D**) Amplification curve and qPCR CT value in CRH X2 site at ZT18; (**E**) Amplification curve and qPCR CT value in CRH N site at ZT22; (**F**) Amplification curve and qPCR CT value in CRH X1 site at ZT22; (**G**) Amplification curve and qPCR CT value in CRH X2 site at ZT22. Values are mean ± SEM, *n* = 6 chickens per time point. *P < 0.05, ** *P* < 0.01, compared with control

To explore the possible link between the site-specific m^6^A modification on *CRH* mRNA and *CRH* mRNA expression in hypothalamus, RNA samples from hypothalamus on ZT18 and ZT22 with significant changes in *CRH* mRNA were subjected to single-base elongation and ligation-based qPCR amplification method (SELECT) assay. Two specific m^6^A sites (Fig. 6A) were identified in the coding sequence (CDS) close to 3’UTR (X1) and 3’UTR (X2) of *CRH* mRNA, respectively, from published MeRIP-seq database [27]. N site located in the 5’UTR without consensus m^6^A motif was selected as a negative control. Chronic CORT exposure did not change the CT value on N site at either ZT 18 (Fig. 6B) or ZT 22 (Fig. 6E), compared with CON group. However, chronic CORT exposure significantly increased (*P* < 0.05) the CT value on both X1 (Fig. 6C, F) and X2 (Fig. 6D, G) at both time points (ZT18 and ZT22), which was in accordance with the significant decrease of *CRH* mRNA in hypothalamus at the same time points.

## Discussion

In this study, we observed that chronic CORT exposure completely abolished the circadian rhythm of plasma melatonin levels in the chicken, indicating a disruption of the endogenous rhythmicity. The effects of CORT on plasma melatonin are biphasic, being stimulatory in the light phase when the melatonin levels are low, while inhibitory in the dark phase when the melatonin levels are high. The avian pineal gland receives circadian input through the release of norepinephrine during the day [37], and the dual effects of CORT on pineal melatonin synthesis are determined by the activation of different adrenoceptors (β or β + α1) during GR activation [38].

The circadian rhythms in birds are controlled by multiple circadian pacemakers in the central nervous system. Here we show, for the first time, the circadian expression of clock genes in chicken hippocampus, hypothalamus, and pituitary. All the 6 core clock genes show circadian rhythms in all the 3 brain areas, although the amplitude and the pattern of oscillation vary among genes and brain areas. It is noted that *BMAL1* oscillates in an opposite pattern from *PER2* and *PER3*, may be because they belong, respectively, to “negative arm” and “positive arm” of the circadian clock gene network [9]. Among 3 brain areas, hypothalamus shows more clear and significant rhythmicity and higher susceptibility to CORT treatment. This agrees with a previous publication that long-term administration of dexamethasone resulted in loss of the expression rhythms in Bmal1 and Clock genes in rat SCN [39]. The mechanisms by which chronic CORT alters the circadian gene expression in the chicken are largely unknown. It is likely that CORT directly regulates clock gene expression through GR-mediated transcriptional regulation [40]. However, as melatonin was reported to play a key role in controlling circadian behavioral responses [41] and the loss of circadian rhythm of plasma melatonin corresponded to the diminished circadian pattern of clock genes in the hypothalamus of CORT-exposed chickens in this study. We speculate that chronic CORT may indirectly affects the expression rhythm of circadian clock gene through alterations in melatonin secretion.

CRH is essential for stress adaptation by mediating HPA axis [1] and involved in the regulation of circadian rhythms [2]. Circadian variations of CRH neuron activity are driven by the SCN and likely mediate the characteristic circadian pattern of HPA axis activity [42]. Chronic unpredictable mild stress induces hyperactivity of HPA axis which is indicated by up-regulation of hypothalamic *CRH* mRNA expression in rats [43]. In contrast, chronic CORT exposure significantly decreased CRH expression in chicken hypothalamus during the dark phase with destroyed circadian rhythms. Many factors contribute to the disparity of the findings, including animal species (nocturnal rats vs. diurnal chickens), stress model, and the time points of the sampling.

Accordingly, genes involved in feeding regulation, including satiety genes *POMC* and *CART* and hunger genes *NPY* and *AgRP* [44], show concerted circadian expression pattern, which is in agreement with a previous report that *AgRP*, *NPY*, *POMC* and *CART* genes are expressed in a circadian rhythm in the hypothalamus [45]. The same as *CRH* and its receptors, the circadian rhythm of these appetite-related genes is also destroyed in chickens subjected to chronic CORT exposure. These CORT-induced alterations in hypothalamic gene expression may associated, at least partly, with the disrupted feeding behavior in the chicken.

The m^6^A methylation plays important roles in the regulation of neurogenesis, circadian rhythm, cognitive function, and stress responses [46]. Here, we provide the first evidence that the global m^6^A level in chicken hypothalamus oscillates in a day, being higher in light phase and lower in dark phase. Interestingly, the circadian rhythm pattern of diurnal chickens is opposite to that reported in nocturnal animals. This makes sense as m^6^A is reported to participate in many stress responses [47], and higher m^6^A level corresponds to higher body activity. However, in this study, chronic CORT exposure disrupted the circadian rhythms of m^6^A methylation levels in hypothalamus. Based on the observation that significant decrease of *CRH* mRNA in the dark phase corresponds to the significant increase in m^6^A levels at the same time points, we speculate that m^6^A may be involved in the post-transcriptional regulation of *CRH* mRNA in chicken hypothalamus. Indeed, the two predicted m^6^A sites X1 and X2 were both hypermethylated at detected time points (ZT18 and ZT22). Therefore, it is likely that the decrease of *CRH* expression was due to m^6^A-mediated mRNA degradation [48]. Nevertheless, a functional verification study is required to elucidate the role of m^6^A on these sites in *CRH* gene regulation in chicken hypothalamus.

## Conclusion

In conclusion, our study shows that chronic CORT exposure eliminated the diurnal patterns of plasma CORT and melatonin levels in the chicken. Hypothalamus is the most susceptible brain region to CORT treatment, as almost all the genes, including clock genes, *CRH*, and feeding-related genes, lost their circadian rhythmicity together with the global m^6^A level. Higher m^6^A on 3’UTR of *CRH* mRNA coincides with lower *CRH* mRNA at night, indicating a possible role of m^6^A in the post-transcriptional regulation of *CRH* expression in chicken hypothalamus. These findings provide evidence of CORT-induced disruption of central circadian rhythmicity in *CRH* expression that leads to dysfunction of HPA axis in the chicken, and also imply a role of RNA m^6^A modification in the regulation of circadian rhythms in the chicken.

## Supplementary Information

**Additional file 1: Fig. S1.** Effect of chronic CORT exposure on inflammation related genes mRNA expression in chicken hypothalamus. (A) *TNF-α*, *IL-1β* and *IL-6* mRNA expression in hypothalamus, and destroyed the circadian rhythms of *TNF-α* and *IL-6* mRNA expression (Fig. S1). The circadian rhythms of inflammation related genes and *TNF-α*, *IL-1β* and *IL-*6 mRNA expression in chicken hypothalamus. (A, D) *TNF-α* gene; (B, E) *IL-1β* gene; (C, F) *IL-6* gene; (D) *TNF-α* gene. The relative mRNA levels of inflammation related genes are normalized to PPIA, *n* = 6 chickens per time point. The data markers in the graphs indicate the inflammation related genes mRNA expression levels, and the results are expressed as the mean ± SEM. The curves represent the 24-h period determined by cosinor analysis. Data from CT2 are double-plotted. *R*^2^ values represent the degree of fitting. **P < 0.01, compared with control.

## Data Availability

Not applicable.

## Abbreviations

AgRP: Agouti-related protein
CART: Cocaine amphetamine-regulated transcript
CORT: Corticosterone
CRH: Corticotropin-releasing hormone
CRHR1: CRH receptors type 1
CRHR2: CRH receptors type 2
Cry: Cryptochrome
GR: Glucocorticoid receptor
HPA: Hypothalamic-pituitary-adrenal
m^6^A: N6-methyladenosine
NPY: Neuropeptide Y
Per: Period
POMC: Proopiomelanocortin
SCN: Suprachiasmatic nucleus
SELECT: Single-base elongation and ligation-based qPCR amplification method
