# Supplementary figures and images for: Chronic corticosterone disrupts the circadian rhythm of CRH expression and m6A RNA methylation in the chicken hypothalamus
Source: J Anim Sci Biotechnol. 2022 Mar 8;13:29. doi: 10.1186/s40104-022-00677-4 (PMC8902767; doi:10.1186/s40104-022-00677-4)

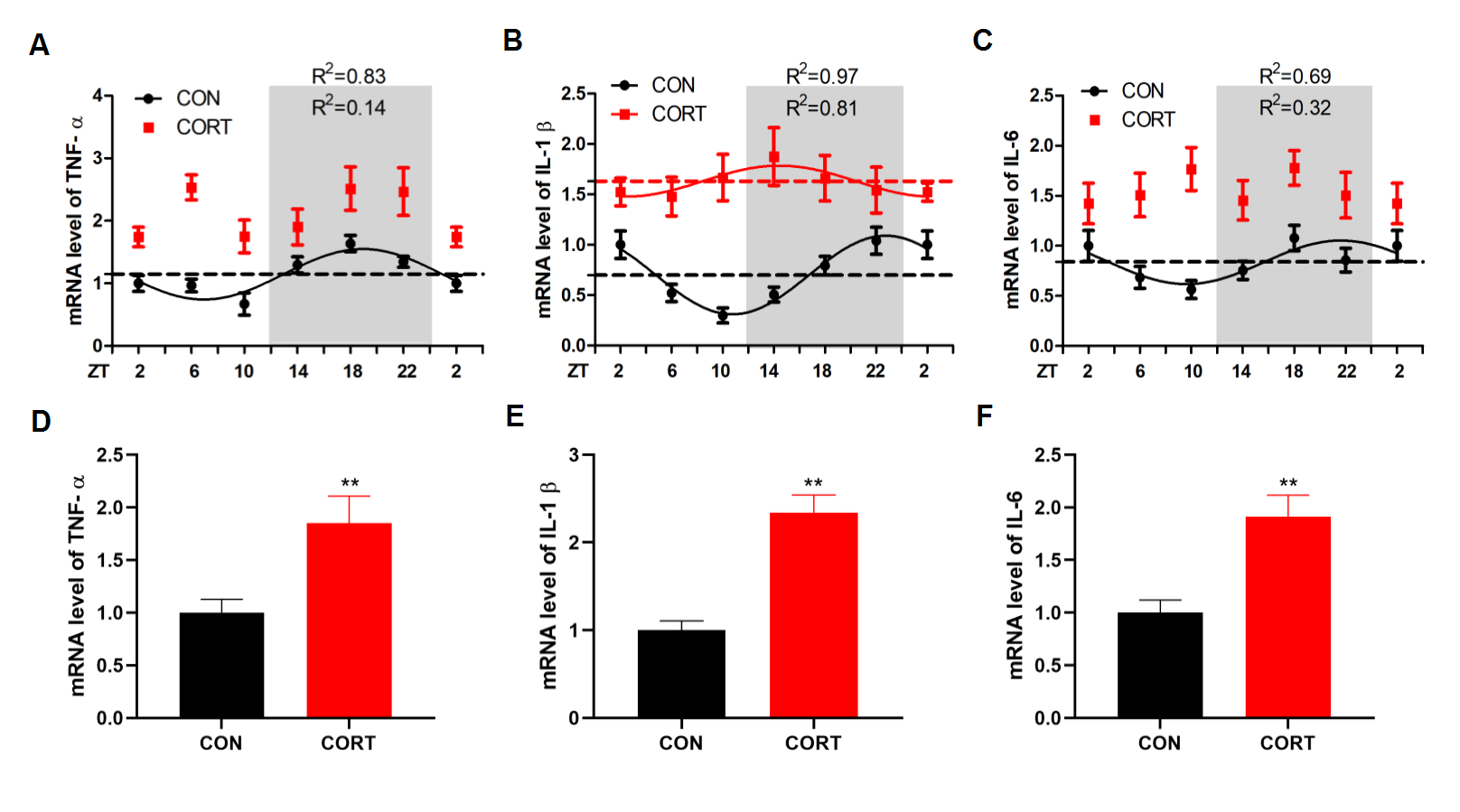

Supplement: Supplementary file 1 — Additional file 1: Fig. S1. Effect of chronic CORT exposure on inflammation related genes mRNA expression in chicken hypothalamus. (A) TNF-α, IL-1β and IL-6 mRNA expression in hypothalamus, and destroyed the circadian rhythms of TNF-α and IL-6 mRNA expression (Fig. S1). The circadian rhythms of inflammation related genes and TNF-α, IL-1β and IL-6 mRNA expression in chicken hypothalamus. (A, D) TNF-α gene; (B, E) IL-1β gene; (C, F) IL-6 gene; (D) TNF-α gene. The relative mRNA levels of inflammation related genes are normalized to PPIA, n = 6 chickens per time point. The data markers in the graphs indicate the inflammation related genes mRNA expression levels, and the results are expressed as the mean ± SEM. The curves represent the 24-h period determined by cosinor analysis. Data from CT2 are double-plotted. R2 values represent the degree of fitting. **P < 0.01, compared with control. [file 40104_2022_677_MOESM1_ESM.tif]
